# NADPH-Cytochrome P450 Reductase Is Regulated by All-*Trans* Retinoic Acid and by 1,25-Dihydroxyvitamin D_3_ in Human Acute Myeloid Leukemia Cells

**DOI:** 10.1371/journal.pone.0091752

**Published:** 2014-03-18

**Authors:** Elżbieta Gocek, Aleksandra Marchwicka, Kamila Bujko, Ewa Marcinkowska

**Affiliations:** Department of Biotechnology, University of Wroclaw, Wroclaw, Poland; University of Kansas Medical Center, United States of America

## Abstract

Acute myeloid leukemia (AML) cell lines can be driven to differentiate to monocyte-like cells by 1,25- dihydroxyvitamin D_3_ (1,25D) and to granulocyte-like cells by all-*trans*-retinoic acid (ATRA). Both compounds activate their specific intracellular receptors, vitamin D receptor (VDR) and retinoic acid receptors (RARs) respectively. Inside the cells 1,25D is degraded to calcitrioic acid by a mitochondrial enzyme CYP24A1, while ATRA is degraded to several polar metabolites by CYP26. NADPH-cytochrome P450 oxidoreductase (POR) is a membrane-bound enzyme required for electron transfer to cytochrome P450 (CYP), vital in the processes of the metabolism of drugs and steroid production in humans. In this paper we report that POR in AML cells, from both cell lines and patients, is upregulated by ATRA and by 1,25D at the level of mRNA and protein. Partial silencing of POR in HL60 cells resulted in augmented differentiation response to 1,25D.

## Introduction

Retinoic acid (RA) and 1,25-dihydroxyvitamin D_3_ (1,25D) are highly active signaling molecules, which regulate many important cellular processes including differentiation [1], [2]. In acute myeloid leukemia (AML) blasts all-*trans*-retinoic acid (ATRA) induces differentiation to granulocyte-like cells, while 1,25D to monocyte-like cells [3]. RA and 1,25D are ligands for specific intracellular receptors, which act as ligand-activated transcription factors. Metabolite of RA, all-*trans*-RA (ATRA) can bind with high affinity to the retinoic acid receptors (RARα, β and γ), while 9-*cis*-RA binds mainly to the retinoid X receptors (RXRα, β and γ) [4]. 1,25D is a ligand to only one vitamin D receptor (VDR), which forms heterodimers with RXRs in its active state [5]. Upon ligation VDR and RARs undergo conformational changes, that allow binding to specific sequences in the promoter regions of target genes, called retinoic acid response elements (RARE) or vitamin D response elements (VDRE).

Since both RA and 1,25D are highly active compounds, their effective concentrations must be strictly regulated in organisms. RA is the biologically active metabolite of either vitamin A or other dietary precursors, such as β-carotene and retinyl palmitate and can't be produced *de novo* by a human body. Since high concentrations of RA are teratogenic and toxic, it's catabolism is strictly regulated [6]. The major catabolizing enzyme is CYP26, whose transcription is upregulated in response to RA [7]. RA-induced transactivation of CYP26 gene is mediated by RARE, which is present in the CYP26 promoter region [8], [9]. Another enzyme, which is located in mitochondria, namely CYP24A1, is responsible for degradation of 1,25D to its inactive metabolite, calcitrioic acid. CYP24A1 is the most strongly regulated VDR-target gene, and its promoter contains multiple VDREs [10]. Cytochrome P450 oxidoreductase (POR) is an electron donor for all microsomal CYP enzymes and functional inactivation of POR is lethal [11]. Embryos of POR deficient mice have significantly elevated levels of RA, what indicates that POR is important for RA catabolism [12]. It has not yet been reported if POR is important for the degradation of 1,25D, however such a role has been previously postulated [13]. Moreover, in our previous studies we found that patients with AML, whose leukemic blasts carry deletion of the whole or part of chromosome 7 are more susceptible to 1,25D-induced differentiation than any other patients [14]. Since the *POR* gene is localized on this chromosome, we hypothesized that lack of POR enzyme might contribute to the higher activity of 1,25D, due to its reduced catabolism. Therefore we investigated if POR gene and/or protein are regulated by ATRA and 1,25D in AML blasts from cell lines and from patients with AML.

## Materials and Methods

### 1. Cell lines

HL60 cells were from the European Collection of Cell Cultures, while KG1 and NB4 cells from German Resource Center for Biological Material (DSMZ GmbH, Braunschweig, Germany). The cells were propagated and kept at standard cell culture conditions [14].

### 2. Chemicals and antibodies

1,25D was purchased from Cayman Europe (Tallinn, Estonia), while ATRA from Sigma-Aldrich (St. Louis, MO). Antibodies CD11b-FITC (cat. No. 21279113), CD14-APC (cat. No. 21270146), CD14-PE (cat. No. 21270144), CD3-PE (cat. No. 21620034), CD20-PE (cat. No. 21279204) as well as appropriately labeled isotype controls were from ImmunoTools (Friesoythe, Germany). Mouse monoclonal anti-POR (cat. No. sc-55477) and rabbit polyclonal anti-actin (cat. No. sc-1616) antibodies were from Santa Cruz Biotechnology Inc. (Santa Cruz, CA). Goat anti-rabbit IgG and anti-mouse IgG conjugated to peroxidase were from Jackson ImmunoResearch (West Grove, PA).

### 3. Isolation of mononuclear cells from peripheral blood

The study was accepted by the local Ethics Committee. The patients were presented to the Department of Hematology, Blood Neoplasms and Bone Marrow Transplantation, Wroclaw Medical University and gave informed consent for this study. 8 ml of peripheral blood was diluted with phosphate-buffered saline (PBS) in 1∶1 ratio. Diluted blood was carefully layered onto an equal volume of LSM 1077 (PAA Laboratories GmbH, Pasching, Austria), and centrifuged at 400×g for 30 min. The opaque interface containing the blast cells was transferred into fresh sterile tubes, and washed three times with PBS. The cells were transferred to RPMI 1640 medium at a density of 10^6^ cells/ml, supplemented with 10% FCS, 100 units/ml penicillin and 100 μg/ml streptomycin and grown in a humidified atmosphere of 95% air and 5% CO2 at 37°C.

### 4. Determination of cell differentiation

The expression of cell surface differentiation markers was determined by flow cytometry. The cells were incubated with 1,25D or ATRA for the desired time and then washed and stained with 1 μl of fluorescently labeled antibody (or the appropriate control immunoglobulins) for 1 h on ice. Next, they were washed three times with ice-cold PBS and suspended in 0.5 ml PBS prior to analysis on FACS Calibur flow cytometer (Becton Dickinson, San Jose, CA). The acquisition parameters were set for an isotype control. Differentiation assays were repeated from 3 to 6 times. In the case of patients' blast cells these were incubated with 1,25D for 96 h, washed with PBS, then incubated with CD14-FITC, CD3-PE, and CD20-PE antibodies. To distinguish between viable and non-viable cells propidium iodide at a final concentration of 0.25 μg/ml was added just before data acquisition. The acquisition parameters were set for appropriate isotypic control. The cells that emitted fluorescence in the red channel (lymphocytes and non-viable cells) were excluded from the analysis. Data analysis was performed with use of WinMDI 2.8 software (freeware by Joseph Trotter) or Flowing Software 2.5.0 (freeware by Perttu Terho).

### 5. Preparation of cell lysates and Western blotting

In order to prepare cytosolic, membrane, nucleosolic and chromatin fractions 5×10^6^ cells/sample (equivalent of 20 μl packed cell volume) were washed with PBS and lysed using Pierce Subcellular Protein Fractionation Kit (Thermo Fisher Scientific Inc., Worcester, MA) according to the user's manual. Obtained lysates were denatured by adding 5x sample buffer (1/4 volume of the lysate) and boiled for 5 min. Mitochondria from 2×10^7^ were isolated using Pierce Mitochondria Isolation Kit for Cultured Cells (Thermo Fisher Scientific Inc., Worcester, MA) according to the user's manual. Obtained mitochondrial pellets were lysed, denatured in 80 μl of 2x sample buffer and boiled for 5 min. 30 μl of lysates were separated in SDS-PAGE and transferred to PVDF membranes, which were incubated successively with primary and a horseradish peroxidase-conjugated secondary antibodies. In order to enhance the reaction for KG1 cells, biotin-conjugated secondary antibody and peroxidase-conjugated streptavidin were used. The protein bands were visualized with a chemiluminescence [15]. The membranes were stripped, and probed with subsequent antibodies. Western blots were repeated 2–4 times.

### 6. cDNA synthesis and Real Time PCR

Total RNA was isolated using TriReagent (Sigma-Aldrich) as per manufacturer's recommendations. RNA quantity was determined using Nanodrop (Thermo Fisher Scientific Inc.) and the quality of RNA was verified by gel electrophoresis. RNA was transcribed into cDNA using High Capacity cDNA Reverse Transcription kit (Applied Biosystems, Foster City, CA). Real Time PCR reaction was performed using SensiFast™ SYBR Hi-ROX Kit (Bioline Reagents Ltd., UK) by Eco Real Time PCR System (Illumina Inc., San Diego, CA). The sequences of POR primers were: forward: 5′-TCTACGACATCGTGGCTGAG-3′ and reverse: 5′-CCAAACACACCCAGGAGACT-3′, and CYP24A1 primers were: forward 5′–CCC ACT AGC ACC TCG TAC C-3′, and reverse 5′–CGT AGC CTT CTT TGC GGT AG-3′. The sequences of GAPDH primers were: forward: 5′-CATGAGAAGTATGACAACAGCCT-3′ and reverse: 5′-AGTCCTTCCACGATACCAAAGT-3′. Real Time PCR assays were repeated 3 times.

### 7. Siliencing of POR gene by shRNA plasmids

Electrotransfection by Neon® Transfection System (Invitrogen™, Carlsbad, CA) was performed as follows: 1×10^6^ cells were transfected with 1 μg of control shRNA plasmid-A (sc-108060, Santa Cruz, CA) or POR shRNA plasmid (sc-35147-SH, Santa Cruz, CA). After transfection the cells were immediately transferred to a 34 mm dish (6 well plate) containing pre-warmed antibiotic free media and incubated at 37 °C. Twenty-four hours after transfection, the cells were washed twice with PBS and resuspended in a selective medium: RPMI medium supplemented with 10% fetal bovine serum (FCS, Sigma, St. Louis, MO), 100 units/ml penicillin, 100 μg/ml streptomycin (Sigma, St. Louis, MO) and 1 μg/ml puromycin (Santa Cruz, CA). Medium and selective antibiotic were changed every 2 days and puromycin non-resistant cells were cleared from the culture.

### 8. Statistical analysis

In order to analyze the results obtained in experiments, a significance of the differences between values was assessed by Student's t-test for independent samples (Excel, Microsoft Office).

## Results

### 1. Differentiation of HL60, KG1 and NB4 cells in response to 1,25D or ATRA

**Figure 1:**
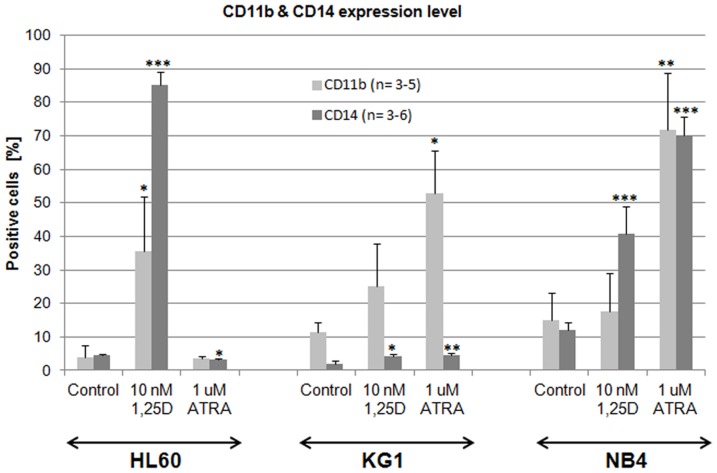
Differentiation of HL60, KG1 and NB4 cells in response to 1,25D and ATRA. The cells were exposed to 1,25D (10 nM) or to ATRA (1 μM) for 96 h and then the expression of differentiation markers CD11b and CD14 was detected using flow cytometry. Mean values (±SEM) of percentages of positive cells are presented in Y-axis. Results that differ significantly from the result for respective control cells are marked with asterisks (*p<0.05; **p<0.01; ***p<0.001).

In our previous paper we demonstrated that various AML cell lines have different susceptibilities to 1,25D- or ATRA-induced differentiation into monocyte-like or granulocyte-like cells respectively [16]. CD11b is a cell adhesion molecule present mostly on the surface of granulocytes and monocytes [17], while CD14 is a co-receptor for bacterial lipopolysaccharide characteristic for monocytes and macrophages [18]. HL60 cell line responded to 1,25D with strong upregulation of CD11b and CD14 cell differentiation markers, while ATRA induced only minor upregulation of CD14. KG1 were almost unresponsive to 1,25D, but responded to ATRA with strong upregulation of CD11b granulocytic differentiation marker [19]. In our current research we added NB4 cells that carry t(15;17), resulting in PML-RARα fusion gene [20]. These cells responded to 1,25D with upregulation of CD14 and to ATRA with upregulation of CD11b and CD14 antigens (Fig. 1).

### 2. Transcription of POR gene in HL60, KG1 and NB-4 cells in response to 1,25D or ATRA

**Figure 2:**
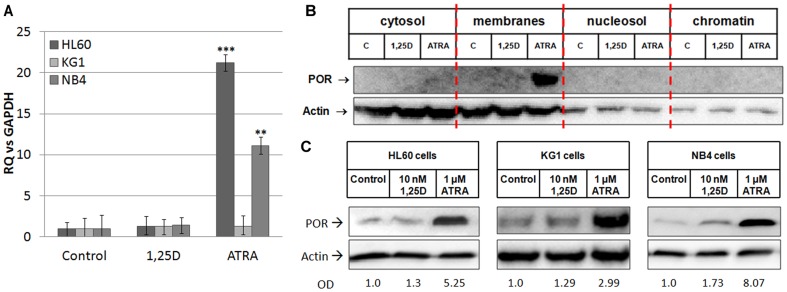
Regulation of *POR* gene and protein by 1,25D and ATRA in AML cells. The cells were exposed to either 1,25D (10 nM) or ATRA (1 μM) and (A) after 96 h the expression of *POR* mRNA was tested in Real Time PCR. The bar charts show mean values (±SEM) of fold changes in *POR* mRNA levels relative to *GAPDH* mRNA levels. The control samples were calculated as 1. Results that differ significantly from the result for respective control cells are marked with asterisks (**p<0.01; ***p<0.001). The expression of POR protein was detected using Western blot in (B) HL60 cells divided into cytosol, membranes, nucleosol and chromatin or (C) in mitochondria of HL60, KG1 and NB4 cells. The proteins were analyzed using anti-POR and anti-actin antibodies. In case of mitochondrial lysates the optical densities (OD) of POR bands divided by ODs of respective actin bands were calculated from 3 blots and the mean values are given below the blots.

Our previous work indicated that patients with AML, whose leukemic blasts carry deletion of the whole or part of chromosome 7 are more sensitive to 1,25D-induced differentiation than any other group of AML patients [14]. We looked at which genes localized on chromosome 7 could contribute to this effect. We hypothesized that the gene encoding POR, an enzyme required for electron transfer to CYP, might be involved in the regulation of the levels of 1,25D. Other studies have shown that lack of POR enzyme results in elevated levels of RA in embryos [12]. Based on this, we decided to investigate if *POR* expression is regulated by either 1,25D or ATRA in the AML cells. The cells were exposed to 10 nM 1,25D or 1 μM ATRA for 96 h, and the expression of *POR* mRNA was tested in Real Time PCR. As presented in Fig. 2A, *POR* mRNA expression was not regulated by 1,25D in any of the cell lines examined. However, when HL60 and NB4 cell lines were exposed to 1 μM ATRA, the expression of *POR* mRNA was significantly upregulated in comparison to the control (p<0.01). It must be noted that upregulation was stronger in HL60 cells than in NB-4 cells. In KG1 cells, *POR* mRNA levels remained similar to that of the control.

In order to further investigate this theory we carried out a preliminary experiment, in which the expression of *POR* gene was studied in mononuclear cells isolated from the peripheral blood of AML patients and compared to the expression in mononuclear cells from healthy donors. The cells were exposed to 10 nM 1,25D or 1 μM ATRA for 96 h, and the expression of both *CYP24A1* and *POR* mRNA was tested in Real Time PCR. Patients' cells exposed for 96 h to 1,25D were also tested by flow cytometry for the expression of CD14 cell differentiation marker. The results for patients' cells are presented in the Table 1. Cells which increased CD14 expression from 1 to 5% in response to 1,25D were marked as (+), between 5 and 10% as (++) and above 10% as (+++). In all patients' samples *CYP24A1* expression was increased after exposure to 1,25D. Since *CYP24A1* is the most strongly regulated VDR-target gene, its increased expression proves that the cells contain transcriptionally active VDR. *POR* expression was increased in all of 1,25D-treated blasts. However, only in two blast samples *POR* expression was upregulated in response to ATRA. In mononuclear cells from all healthy donors *CYP24A1* expression was increased after exposure to 1,25D, showing that VDR protein is transcriptionally active. However in the majority of healthy cells POR was not upregulated in response to either 1,25D or ATRA (Table 2).

**Table 1:** Responses of AML patient's mononuclear cells to 1,25D or ATRA.

Patient			Differentiation	CYP24A1 expression	POR expression	
No	AML subtype	Fusion gene/oncogene	increased CD14	1,25D-induced	1,25D-induced	ATRA-induced
9/2012	M4	FLT3-ITD	+++	14 100 x	5.7 x	2.5 x
12/2012	M1	-	-	41 900 x	2.6 x	1.7 x
13/2012	M1	-	+	14 700 x	1.7 x	downregulation
3/2013	M5b	FLT3-ITD	+++	8 720 x	1.4 x	downregulation
4/2013	M4	CBFB-MYH11	++	24 620 x	58 x	downregulation

Cells from peripheral blood of AML patients were exposed for 96 h to 10 nM 1,25D or to 1 μM ATRA. Then the expression of CD14 antigen in 1,25D-treated blast cells was studied in flow cytometry. From remaining 1,25D-treated blasts and from ATRA-treated blasts mRNA was isolated and the expression of *CYP24A1* and *POR* genes was studied in Real Time PCR.

**Table 2:** Responses of mononuclear cells from healthy donors to 1,25D or ATRA.

Healthy	CYP24A1 expression	POR expression	
No	1,25D-induced	1,25D-induced	ATRA-induced
1/2014	2 633 x	downregulation	downregulation
2/2014	6 109 x	downregulation	1.9 x
3/2014	4 713 x	downregulation	downregulation
4/2014	5 075 x	downregulation	downregulation
5/2014	2 163 x	downregulation	1.05 x

Cells from peripheral blood of healthy donors were exposed for 96 h to 10 nM 1,25D or to 1 μM ATRA. Then the mRNA was isolated and the expression of *CYP24A1* and *POR* genes was studied in Real Time PCR.

### 3. POR protein in membranes and in mitochondria of the cells exposed to 1,25D or ATRA

In vertebrates, two principal types of mono-oxygenases and electron transfer chains have been identified. One is located in the endoplasmic reticulum and the other one in the mitochondrial inner membrane [21]. POR is a membrane-bound enzyme and was reported to be present in an endoplasmic reticulum [13]. Therefore we first studied if we could detect POR protein in the lysates produced either from whole cells or from the cytoplasmic fraction. However, in these lysates the antibody recognized multiple bands of variable molecular masses, but not the correct one (not shown). Then we fractionated the HL60 cells exposed to either 10 nM 1,25D or 1 μM ATRA for 72 h into cytosol, membranes, nucleosol and chromatin and used Western blotting to detect POR. As presented in Figure 2B, a very strong signal corresponding to POR protein was detected in membrane fraction of HL60 cells exposed to ATRA. However, past studies revealed some subcellular localizations of cytochromes P450 and also of their reductases in other cellular membranes [22], [23]. Therefore we decided to check the subcellular fraction of mitochondria. For this purpose the mitochondria were isolated from HL60, KG1 and NB4 cells exposed to either 10 nM 1,25D or 1 μM ATRA for 96 h. It this case detection with anti-POR antibody revealed one band at the level corresponding to the molecular mass of POR (about 80 kDa). It should be noted that in order to visualize POR protein in KG1 cells the biotin-streptavidin system was used to enhance the Western blotting reaction. Enzyme expression in the mitochondria appeared to be slightly elevated after exposure of the cells to 1,25D and particularly after ATRA treatment in all studied cell lines (Fig. 2C).

### 4. Results of POR silencing in HL60 cells

**Figure 3:**
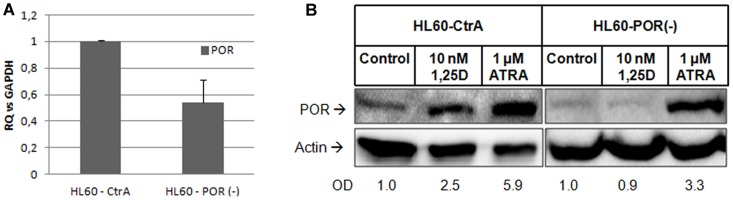
Expression of *POR* gene and protein in transfected HL60 cells. HL60 cells were transfected with plasmids containing either control shRNA (CtrA) or *POR* shRNA and then (A) the expression of *POR* mRNA was tested in Real Time PCR. The bar charts show mean values (±SEM) of fold changes in mRNA levels relative to *GAPDH* mRNA levels. The control sample was calculated as 1. (B) The expression of POR protein in mitochondria of the cells exposed to 1,25D (10 nM) or ATRA (1 μM) for 96 h was detected using Western blot. Mitochondrial proteins were analyzed using anti-POR and anti-actin antibodies. Optical densities (OD) of POR bands divided by ODs of respective actin bands were calculated from 4 blots and mean values are given below the blots.

**Figure 4:**
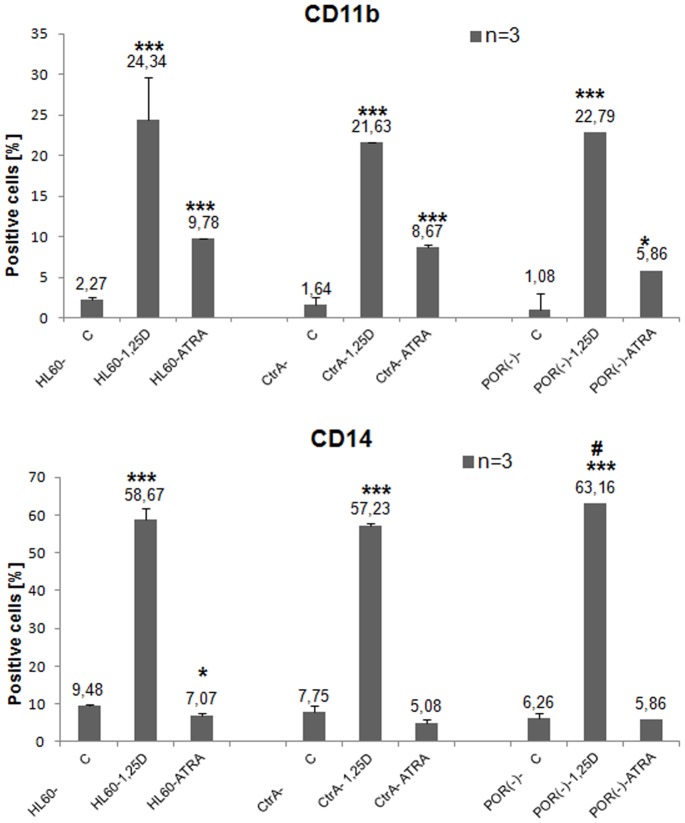
Differentiation of transfected HL60 cells exposed to 1,25D or ATRA. HL60 cells were transfected with plasmids containing either control shRNA (CtrA) or *POR* shRNA and then were exposed to either 1,25D (10 nM) or ATRA (1 μM) for 96 h. The expression of differentiation markers CD11b (upper graph) and CD14 (bottom graph) was detected using flow cytometry. Mean values (±SEM) of percentages of positive cells are presented in Y-axis. Results that differ significantly from the result for respective control cells are marked with asterisks (*p<0.05; ***p<0.001), while the sample which differs from the appropriate CtrA sample is marked with hash (^#^p<0.05).

Our studies documented that POR protein is highly upregulated in mitochondria of HL60 and NB4 cells by ATRA and to some extent by 1,25D, whilst *POR* gene expression is upregulated only by ATRA. Therefore we wanted to study if this enzyme has any effect on the differentiation induced by these two compounds. In order to do this we tried to silence *POR* gene in the above cells. However, the electroporation procedure appeared to be harmful to both NB4 and KG1 cells. Thus, only HL60 cells were transfected with 1 μg of plasmids containing either control shRNA (CtrA) or *POR* shRNA. For the verification of silencing efficiency, we determined *POR* gene expression level in the Real Time PCR, using GAPDH as a reference gene. It appeared that shRNA silenced the *POR* gene expression to the level of 54% in comparison to CtrA cells (Fig. 3A). Since *POR* silencing was not complete at the mRNA level, we wanted to verify, if this procedure had any effect on protein level. For this purpose the transfected HL60 cells were exposed to either 10 nM 1,25D or 1 μM ATRA for 96 h and Western blot analysis was performed to detect the presence of POR protein in mitochondria. We observed that in the cells exposed to 1,25D there was no increase in POR level above the untreated sample, while in the cells exposed to ATRA the POR level increased less (to about 56%) than in the CtrA cells (Fig. 3B). In order to verify if reduced POR enzyme level may contribute to the changed activities of either 1,25D or ATRA, the transfected HL60 cells were exposed to these compounds for 96 h and then the expression of differentiation markers was examined by flow cytometry. As presented in Figure 4, the expression of CD11b was not affected by *POR* silencing in HL60 cells exposed either to 1,25D or to ATRA. However, the expression of CD14 surface antigen was slightly, but significantly elevated in silenced cells exposed to 1,25D, but not to ATRA in comparison to the appropriate CtrA sample (Fig. 4).

## Discussion

The network of various CYP enzymes is very important for activation and degradation of multiple steroids. These enzymes are heme-binding monooxygenases responsible for oxidative conversions of steroid hormones. The major enzyme responsible for ATRA activation is lecithin:retinol acyltransferase (LRAT), which does not belong to the CYP superfamily [4], but the major catabolizing enzymes are members of CYP26 subfamily, where different isoforms of CYP26 have different preferences for RA isomers [6]. In the case of vitamin D, the activation and degradation enzymes belong to the CYP superfamily. The two steps of activation are maintained primarily by CYP27A1 or CYP2R1 (25-hydroxylation) and subsequently by CYP27B1 (1α-hydroxylation), while all steps of catabolism from 1,25D to calcitrioic acid are executed by CYP24A1 [24]. The homeostasis of 1,25D and RA is maintained by negative feedback loops, since expressions of CYP26A1 and CYP24A1 are strictly regulated by ATRA and 1,25D respectively. Mutations or inactivation of POR (an electron donor for all microsomal CYP enzymes) have detrimental effects to the organism. Knockout of *POR* gene is early embryonic lethal in mice [11], while mutations and polymorphisms in humans are connected with disordered steroidogenesis, congenital adrenal hyperplasia, Antley-Bixler or Williams syndromes [25], [26], [27]. Since RA activation does not depend on CYP enzymes, and its degradation does, it is not surprising that *POR^-/-^* mice embryos have overall elevated levels of RA [11]. The above data led us to question, whether or not the *POR* gene itself is a target of a regulation by either ATRA or 1,25D.

The models used in our studies were AML cells, which in response to ATRA differentiate to granulocyte-like, while in response to 1,25D to monocyte-like (Fig. 1). Since the differentiation effects may be measured in quantitative ways, these models might allow us to measure the effects of *POR* silencing. The first model, HL60 cells, are strongly responsive to 1,25D, and slightly to ATRA and have high expression levels of VDR mRNA and protein [16]. The second model, KG1 cells, are responsive to ATRA, but resistant to the actions of 1,25D, due to a very low expression level of VDR [16]. Finally, for the third model we used NB4 cells, where the RARα receptor is mutated [20]. In HL60 and NB4 cells the expression of *POR* mRNA was upregulated by ATRA, however the level in NB4 cells was lower than in HL60 cells (Fig. 2A). This effect suggests that various isoforms of RAR proteins are involved in the upregulation of POR transcription. Interestingly, in KG1 cells ATRA did not influence *POR* expression, suggesting that VDR may also participate in the upregulation. From the five samples of patients' AML blasts, only two responded to ATRA with upregulation of *POR* gene, and the extent of upregulation was lower than in the cell lines (Table 1). This might suggest that the isoforms of RAR responsible for regulation of *POR* might not be present as proteins in the blasts of non responding patients. However, in order to verify this mechanism further studies need to be conducted using a bigger group of patients. We did not observe any effect on *POR* mRNA when the cell lines and mononuclear cells from healthy donors were exposed to 1,25D (Table 2), but all patients' blasts responded to 1,25D with upregulation of *POR* gene (Table 1). However, further experiments showed that not only transcription regulates the levels of POR enzyme in the cells. The POR protein was not present in membrane fraction of untreated HL60 cells and it increased significantly after exposure of these cells to ATRA (Fig. 2B). Additionally in HL60 and NB4 cells exposed to ATRA we observed a very strong increase of POR protein levels in mitochondria, but also some increase in the cells exposed to 1,25D. Significant, however not as strong as in other cell lines an increase of POR protein in mitochondria was observed in KG1 cells (Fig. 2C). The above results suggest that there are some additional to transcriptional regulation mechanisms of POR levels regulation, possibly increased stability of protein or of mRNA encoding it. Next we decided to silence the expression of *POR* gene in the AML cells. In general, AML cells are difficult to transfect, and transfection using siRNA appeared to be inefficient (not shown). Because of this we decided to use shRNA, and further select the transfected cells using the appropriate selection antibiotic. It appeared that only HL60 were possible to transfect, and only with the electroporation procedure. The cells were transfected with a control plasmid (CtrA) and with a plasmid encoding shRNA against *POR*. The silencing appeared to be partial, as the gene was downregulated by approximately 50% at the mRNA level (Fig. 3A). Upon studying the protein levels in the silenced cells exposed to 1,25D, it appeared that POR levels did not rise over untreated control, however when the cells were exposed to ATRA, the POR levels increased, but this increase was less than in the cells transfected with the control plasmid (Fig. 3B). Finally we investigated if POR has any effect on either ATRA or 1,25D-induced cell differentiation. Therefore we studied the differentiation effect of both compounds in transfected HL60 cells. The statistically significant increase in a CD14 cell differentiation marker was observed only when the cells with silenced *POR* were exposed to 1,25D, but not to ATRA (Fig. 4). It is likely that we saw no effect in ATRA-treated cells due to the fact that *POR* silencing was not complete, and the increase of the POR levels in cells treated with ATRA was still high enough, so that the enzyme level was sufficient to degrade ATRA added to the culture.

Previous research has shown that POR is an enzyme important for the maintenance of steroid hormones at the level of the whole organism. Its involvement in regulation of global levels of retinoic acid metabolites was documented [11], while its involvement in regulation of 1,25D levels was postulated [13]. In this study we have shown for the first time that *POR* gene and POR protein content are regulated in AML cells by ATRA and by 1,25D. We have also observed that POR protein is present not only in the membrane fraction which contains endoplasmic reticulum, but also in the mitochondria, where it could participate in 1,25D catabolism. These observations suggest that POR might be important for maintaining local intracellular levels of ATRA and 1,25D. Since these two compounds are important for myeloid cell differentiation, the disturbances in POR activity or expression levels might contribute to the cancer phenotype of myeloid cells. Larger studies using AML patients' cells and their comparison to normal blood cells, preferably in mice models, are necessary to investigate this hypothesis.
